# Supplementary material for: Modeling glioblastoma heterogeneity as a dynamic network of cell states
Source: Mol Syst Biol. 2021 Sep 16;17(9):e10105. doi: 10.15252/msb.202010105 (PMC8444284; doi:10.15252/msb.202010105)
Supplement: Supplementary file 6 — Source Data for Figure 5 [file MSB-17-e10105-s004.zip › Figure5A_sourcedata/GSEA_3071/hallmarks_stateIV.GseaPreranked.1623742551702/gsea_report_for_na_pos_1623742551702.html]

Report for na\_pos 1623742551702 [GSEA]

| GS  follow link to MSigDB | GS DETAILS | SIZE | ES | NES | NOM p-val | FDR q-val | FWER p-val | RANK AT MAX | LEADING EDGE || 1 | HALLMARK\_KRAS\_SIGNALING\_DN | Details ... | 32 | 0.46 | 1.98 | 0.002 | 0.006 | 0.019 | 943 | tags=47%, list=18%, signal=57% |
| 2 | HALLMARK\_PANCREAS\_BETA\_CELLS | Details ... | 15 | 0.49 | 1.65 | 0.036 | 0.046 | 0.255 | 1417 | tags=67%, list=27%, signal=91% |
| 3 | HALLMARK\_KRAS\_SIGNALING\_UP | Details ... | 75 | 0.28 | 1.42 | 0.044 | 0.145 | 0.763 | 653 | tags=29%, list=12%, signal=33% |
| 4 | HALLMARK\_TGF\_BETA\_SIGNALING | Details ... | 34 | 0.26 | 1.10 | 0.335 | 0.534 | 1.000 | 1091 | tags=41%, list=21%, signal=52% |
| 5 | HALLMARK\_HEME\_METABOLISM | Details ... | 70 | 0.18 | 0.90 | 0.617 | 0.886 | 1.000 | 767 | tags=23%, list=14%, signal=26% |
| 6 | HALLMARK\_OXIDATIVE\_PHOSPHORYLATION | Details ... | 78 | 0.11 | 0.56 | 0.991 | 1.000 | 1.000 | 765 | tags=14%, list=14%, signal=16% |
| 7 | HALLMARK\_NOTCH\_SIGNALING | Details ... | 16 | 0.12 | 0.41 | 0.995 | 0.998 | 1.000 | 785 | tags=19%, list=15%, signal=22% |
Table: Gene sets enriched in phenotype **na **[plain text format]****
